# Supplementary material for: The Successful Treatment of a Patient with Ehlers–Danlos Syndrome (EDS) After an Extensive Burn Injury: A Case Report
Source: Medicina (Kaunas). 2025 Mar 21;61(4):554. doi: 10.3390/medicina61040554 (PMC12028372; doi:10.3390/medicina61040554)
Supplement: Supplementary file 1 [file medicina-61-00554-s001.zip › medicina-3463054-supplementary.pdf]

Table S1. Summary of clinical subtypes of EDS [1].

Clinical EDS subtype	Inheritance pattern	Main criteria	Secondary criteria
Classical EDS	Autosomal dominant	1. Excessive stretching of the skin and atrophic scars<br>2. Generalized joint hypermobility<br>(GJH, generalized joint hypermobility)	1. Easy bruising<br>2. Soft, supple skin<br>3. Subcutaneous spheroids<br>4. Hernia<br>5. Joint hypermobility (e.g. sprains, dislocations/<br>subluxation, pain, elastic flat feet)<br>6. Family history (relative who meets the clinical criteria
Classical-like EDS	Autosomal recessive	1. Excessive stretching of the skin, with velvety texture and lack of atrophic scar<br>2. Dislocations (most often of the shoulder and ankle)<br>3. Skin that is prone to bruising or spontaneous bruising petechiae	1. Foot deformities: wide forefoot, brachydactyly; flat foot; hallux valgus; piezogenic papules<br>2. Swelling of the legs in the absence of myocardial infarction<br>3. Proximal and distal muscle weakness<br>4. Axonal polyneuropathy<br>5. Muscle atrophy of the hands and feet<br>6. Acrogeric hands, clinodactyly, brachydactyly<br>7. Vaginal/uterus/rectal prolapse<br>Confirmatory molecular tests are necessary to make a definitive diagnosis.
Cardiac-Valvular EDS (cvEDS)	Autosomal recessive	1. Severe progressive valvular heart disease<br>2. Skin: hyperextensibility of the skin, atrophic scars, thin skin, easily formed bruises<br>3. Hypermobility of the joints (generalized or<br>limited to small ponds)	1. Inguinal hernia<br>2. Chest deformity<br>3. Joint dislocations<br>4. Foot deformities:<br>flat feet, hallux valgus<br>• Minimum criteria for suggesting a cvEDS:<br>– Main criterion: severe, progressive problems with the heart valves<br>– AND a compatible family history with autosomal recessive inheritance<br>Plus<br>– Or: one other main criterion<br>– And/or: at least two secondary criteria.<br>Confirmatory molecular tests are necessary for a definitive diagnosis
Vascular EDS (vEDS)	Autosomal recessive	1. Family history of vEDS (documented causative variant in COL3A1)<br>2. Rupture of an artery at a young age	1. Bruising not related to the identified trauma and/or in unusual places like cheeks and back<br>2. Thin, transparent skin with increased visibility of veins<br>3. Distinctive facial appearance<br>4. Spontaneous pneumothorax

		3. Spontaneous perforation of the sigmoid colon in the absence of a known cause diverticular disease or other bowel pathology<br>4. Rupture of the uterus during the third trimester (in the absence of caesarean section and/or severe perinatal period in a previous pregnancy)<br>5. Carotid cavernous sinus fistula (CCSF)	5. Acrogeria<br>6. Talipes equinovarus<br>7. Congenital hip dislocation<br>8. Hypermobility of small joints<br>9. Tendon and muscle rupture<br>10. Keratoconus<br>11. Gum brittleness<br>12. Early-onset varicose veins (under age 30 and nulliparous)
Hypermobile EDS (hEDS)	Autosomal recessive	The diagnosis of hEDS is clinical. There is no exist noteworthy data on the genetic etiology of the disease.	
Arthrchalasia EDS (aEDS)	Autosomal dominant	1. Congenital bilateral hip dislocation<br>2. Heavy GJH, with numerous dislocations<br>3. Excessive stretching of the skin	1. Kyphoscoliosis<br>2. Radiologically benign osteopenia<br>3. Tissue fragility, including atrophic scars<br>4. Bruise-prone skin<br>• Minimum criteria suggesting aEDS:<br>– Main criterion: Congenital bilateral hip dislocation<br>Plus<br>– One of the main criteria: hyperextensibility of the skin<br>– Or main criterion: heavy GJH with multiple dislocations and at least two other criteria. Confirmatory molecular tests are necessary to make a definitive diagnosis.
Dermatosparaxia EDS (dEDS)	Autosomal recessive	1. Extreme skin fragility with congenital or postpartum skin tears<br>2. Characteristic features of the facial skeleton, which are visible at birth or early infancy<br>3. Flabby skin, with excessive skin folds<br>4. Enlarged hand wrinkles<br>5. Severe bruising with the risk of subcutaneous hematomas<br>6. Umbilical hernia<br>7. Postpartum growth retardation<br>8. Short limbs<br>9. Perinatal complications due to fragility of connective tissue	1. Soft and supple skin<br>2. Atrophic scars<br>3. GJH<br>4. Visceral fragility<br>(e.g. bladder rupture, diaphragm rupture, rectal prolapse)<br>5. Delayed motor development<br>6. Osteopenia<br>7. Hirsutism<br>8. Abnormal tooth development<br>9. myopia, strabismus, astigmatism,<br>• Minimum criteria suggesting dEDS:<br>– Main criterion: extreme skin fragiity<br>– AND the main criterion: characteristic features of the facial skeleton<br>Plus<br>– one other primary criterion or three secondary criteria.<br>Confirmatory molecular tests are necessary to make a definitive diagnosis.
Kyphoscolyotic (kEDS)	Autosomal recessive	1. Congenital muscular hypotension	1. Excessive stretching of the skin<br>2. Bruise-prone skin

		2. Congenital or early kyphoscoliosis<br>3. GJH with sprains (shoulders, hips and knees)	3. Rupture/aneurysm of a medium-sized artery<br>4. Osteopenia/osteoporosis<br>5. Blue sclera<br>6. Hernia (umbilical or inguinal)<br>7. Chest deformity<br>8. Marfan syndrome<br>9. Clubfoot<br>10. Nearsightedness, hyperopia<br>• Gene-specific secondary criteria for PLOD1 gene:<br>1. Skin fragility (poor wound healing, enlarged atrophic scars)<br>2. Fragility of the sclera and eyeball<br>3. Microcornea<br>4. Facial dysmorphia
Fragile corneal syndrome (BCS)	Autosomal recessive	1. Thin cornea, with or without rupture<br>2. Keratoconus<br>3. Blue sclera	1. Corneal scarring as Result of rupture<br>2. Progressive loss of corneal stroma<br>3. Myopia<br>4. Retinal detachment<br>5. Deafness (tonal audiogram "sloping")<br>6. Hip dysplasia<br>8. Hypotension in infants, usually mild<br>9. Scoliosis<br>10. Arachnodactyly (finger spidering)<br>11. GJH<br>13. Benign finger contractures<br>14. Soft velvety leather, transparent skin
Spondylodyplastic EDS (spEDS)	Autosomal recessive	1. Short stature<br>2. Muscular hypotension<br>3. Limb laxity	1. Hyperextensibility of the skin, thin translucent skin<br>2. Platypodia<br>3. Delayed motor development<br>4. Osteopenia<br>5. Delayed cognitive development<br>• Gene-specific secondary criteria for B4GALT7 gene:<br>– Radioulnar synostosis<br>– Bilateral contractures of the elbow or limited elbow movement<br>for GJH32 gene:<br>– Single transverse palmar fold<br>– Severe hypermetropia<br>– Cloudy cornea<br>for B3GALT6 gene:<br>– Kyphoscoliosis<br>– Hypermobility of the joints, generalized or limited to the distal joints<br>– Peculiar fingers (arachnodactyly, spatulas)

			– Tooth discoloration, dysplastic teeth<br>B4GALT7 include:<br>triangular face, wide-set eyes, exophthalmos, narrow lips, low-set ears, sparse hair on the head, abnormal dentition, flat face, wide forehead, blue sclera and cleft palate.<br>– Osteoporosis with multiple spontaneous fractures<br>– Aneurysm of the ascending aorta<br>SLC39A13:<br>– Bulging eyes<br>– Atrophy of the glomerular muscles and tapered fingers
Musculoskeletal EDS (msEDS)	Autosomal recessive	1. Congenital multiple contractures, metatarsus varus<br>2. Characteristic features of the facial skeleton, which are visible at birth or in the early period of infancy<br>3. Characteristic features of the skin, including overstretching, easy bruising	1. Recurrent/chronic sprains<br>2. Chest deformities<br>3. Spinal deformities<br>5. Progressive foot deformities<br>6. Large subcutaneous hematomas<br>7. Chronic constipation<br>8. Diverticula of the colon<br>9. Pneumothorax/pneumothorax<br>10. Nephrolithiasis<br>11. Hydronephrosis<br>12. Cryptorchidism in males<br>14. Myopia, astigmatism, glaucoma
Periodontal EDS (pEDS)	Autosomal dominant	1. Severe and incurable periodontitis with an early onset<br>2. Cleft palate<br>3. Family history of a first-degree relative who meets clinical criteria	1. Hyperextensibility, abnormal scarring<br>2. Increased number of infections<br>3. Hernia<br>4. Marfanoid facial features<br>5. Pronounced vascularity<br>• Minimum criteria for suggesting pEDS:<br>– Main criterion (1): serious and Early-onset incurable periodontitis OR main criterion (2): cleft palate<br>Plus<br>Confirmatory molecular tests are necessary to make a definitive diagnosis.
Myopatic EDS	Autosomal dominant or	1. Congenital muscular hypotension<br>2. Contractures of the proximal joints (knee,hip and elbow)	1. Atrophic scars<br>2. Delay in motor development<br>3. Myopathy in muscle biopsy<br>• Minimum clinical criteria suggesting medications:

	Autosomal recessive	3. Hypermobility of the distal joints	– Main criterion: congenital muscle hypotension that improves with age<br>Plus<br>– Or: one other main criterion<br>– And/or: three secondary criteria.<br>Confirmatory molecular tests are necessary for a definitive diagnosis

Table S2. List of clinical subtypes of EDS [1].

Clinical EDS subtype	Abbreviation	Inheritance pattern	Responsible Gene	Protein
Classical EDS	cEDS	Autosomal dominant	Major: COL5A1,<br>Rare: COL1A1	Type V collagen<br>Type I collagen
Cardiac-valvular	cvEDS	Autosomal recessive	COL1A2	Type I collagen
Musculocontractural EDS	mcEDS	Autosomal recessive	CHST14	D4ST1
Dermatosparaxis EDS	dEDS	Autosomal recessive	ADAMTS2	ADAMTS-2
Kyphoscollotic EDS	kEDS	Autosomal recessive	PLOD1<br>FKBP14	LH1<br>FKBP22
Periodontal EDS	pEDS	Autosomal dominant	C1R<br>C1S	C1r<br>C1s
Brittle Cornea syndrome	BCS	Autosomal recessive	ZNF469	ZNF469
